# Fat embolism syndrome after nailing an isolated open tibial fracture in a stable patient: a case report

**DOI:** 10.1186/1756-0500-7-237

**Published:** 2014-04-14

**Authors:** Gustavo Aparicio, Isabel Soler, Luis López-Durán

**Affiliations:** 1 Department of Orthopaedic Surgery, San Carlos University Hospital, Complutense University, c/Profesor Martín Lagos Avenue s/n, Madrid 28040, Spain

**Keywords:** Fat embolism syndrome, Open tibial fracture, Intramedullary nailing

## Abstract

**Background:**

Fat embolism syndrome is a potentially fatal complication of long bone fractures. It is usually seen in the context of polytrauma or a femoral fracture. There are few reports of fat embolism syndrome occurring after isolated long bone fractures other than those of the femur.

**Case presentation:**

We describe a case of fat embolism syndrome in a 33-year-old Caucasian man. He was being seen for an isolated Gustilo’s grade II open tibial fracture. He was deemed clinically stable, so we proceeded to treat the fracture with intramedullary reamed nailing. He developed fat embolism syndrome intraoperatively and was treated successfully.

**Conclusion:**

This case caused us to question the use of injury severity scoring for isolated long bone fractures. It suggests that parameters that have been described in the literature other than that the patient is apparently clinically stable should be used to establish the best time for nailing a long bone fracture, thereby improving patient safety.

## Background

Fat embolism syndrome (FES) is a potentially fatal complication (mortality 10-36) [1,2] of long bone fractures. Classically described as the triad of hypoxia, petechiae, and neurological impairment, it is characterized by bone marrow fat entering the systemic circulation and the individual’s inflammatory response to it. The response can result in dysfunction of several organs, most importantly the lungs, brain, and skin. Although fat embolization occurs in the majority of patients with long bone fractures or during orthopedic procedures, clinical signs and symptoms occur in only 1-10% of these patients [2,3]. Most of the reported cases occurred in patients with multiple traumatic injuries that resulted in the systemic inflammatory response syndrome, which causes multi-organ damage via a reaction to free fatty acids [2].

There are few reports of FES occurring after isolated long bone fractures other than in patients with a femoral fracture [4]– [7]. There is no consensus as yet regarding the most appropriate method of nailing these fractures or on the timing of the fixation to minimize the incidence of FES [8].

Our aim was to report a case of FES in a seemingly otherwise healthy patient who sustained an isolated open fracture of the tibia. There were no factors present that would predispose the patient to FES. Hence, the fracture was treated with reamed intramedullary nailing.

## Case presentation

**Figure 1:**
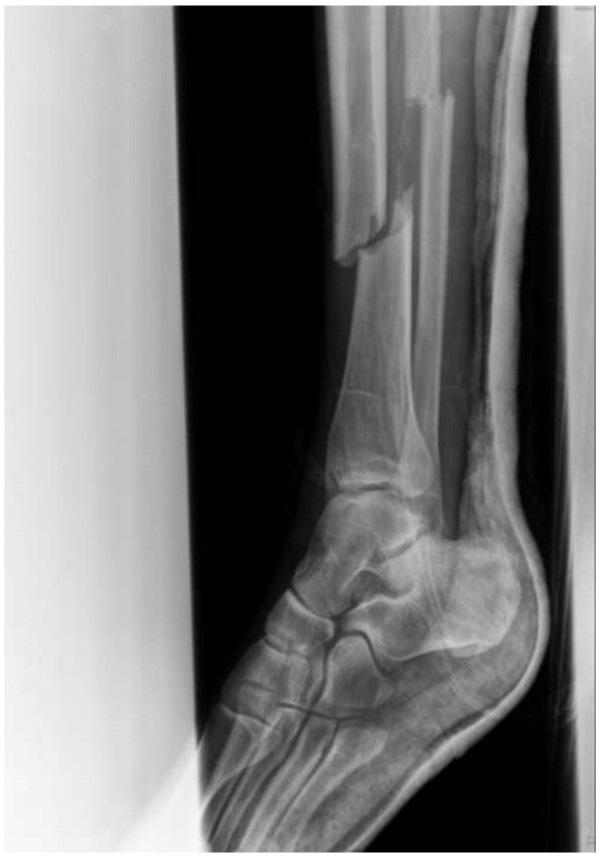
Radiograph of the left tibia at presentation in the emergency room.

**Figure 2:**
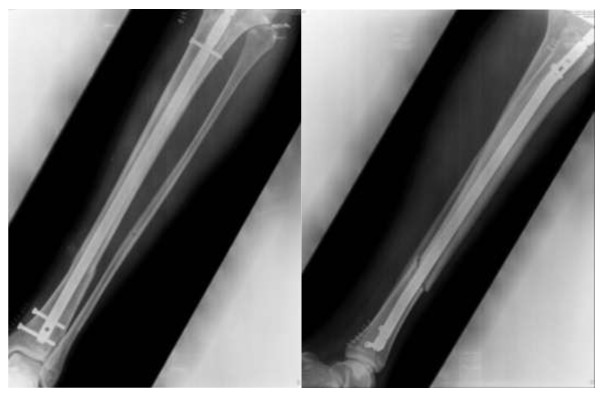
Anteroposterior and lateral radiographs of the left tibia after nailing.

A 33-year-old healthy Caucasian male skier was transferred to our institution 48 hours after a ski accident. His left leg had been temporarily fixed with a plaster cast. The wound had been debrided, irrigated, and closed at a local ski resort hospital. A Gustilo grade II open left tibial fracture (42-A2.2 according to the Orthopaedic Trauma Association) [9,10] was diagnosed (Figure 1). Reevaluation of the patient showed a Glasgow Coma Scale of 15, an Injury Severity Score (ISS) [11] of 9, a New Injury Severity Score (NISS) [12] of 9, and unremarkable chest radiography (Figure 2). No other sites of injury were identified.

**Figure 3:**
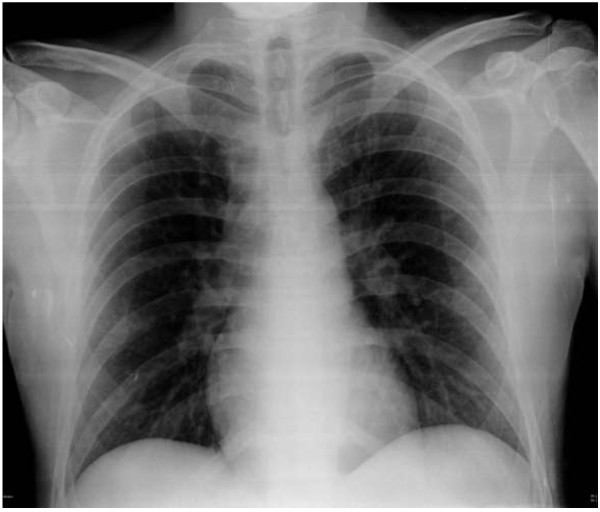
Normal preoperative anteroposterior chest radiograph.

**Figure 4:**
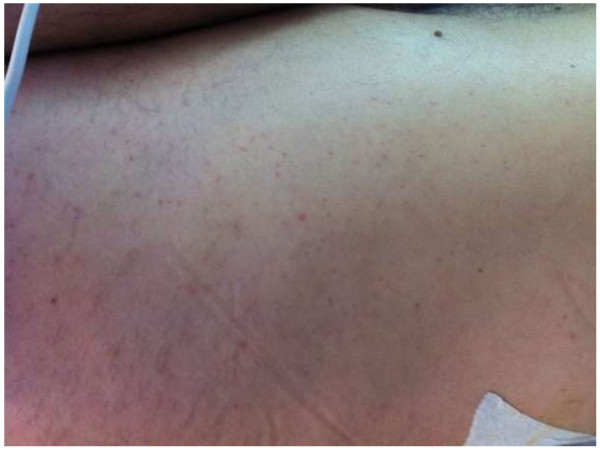
Petechiae on the patient’s lateral chest and abdomen.

**Figure 5:**
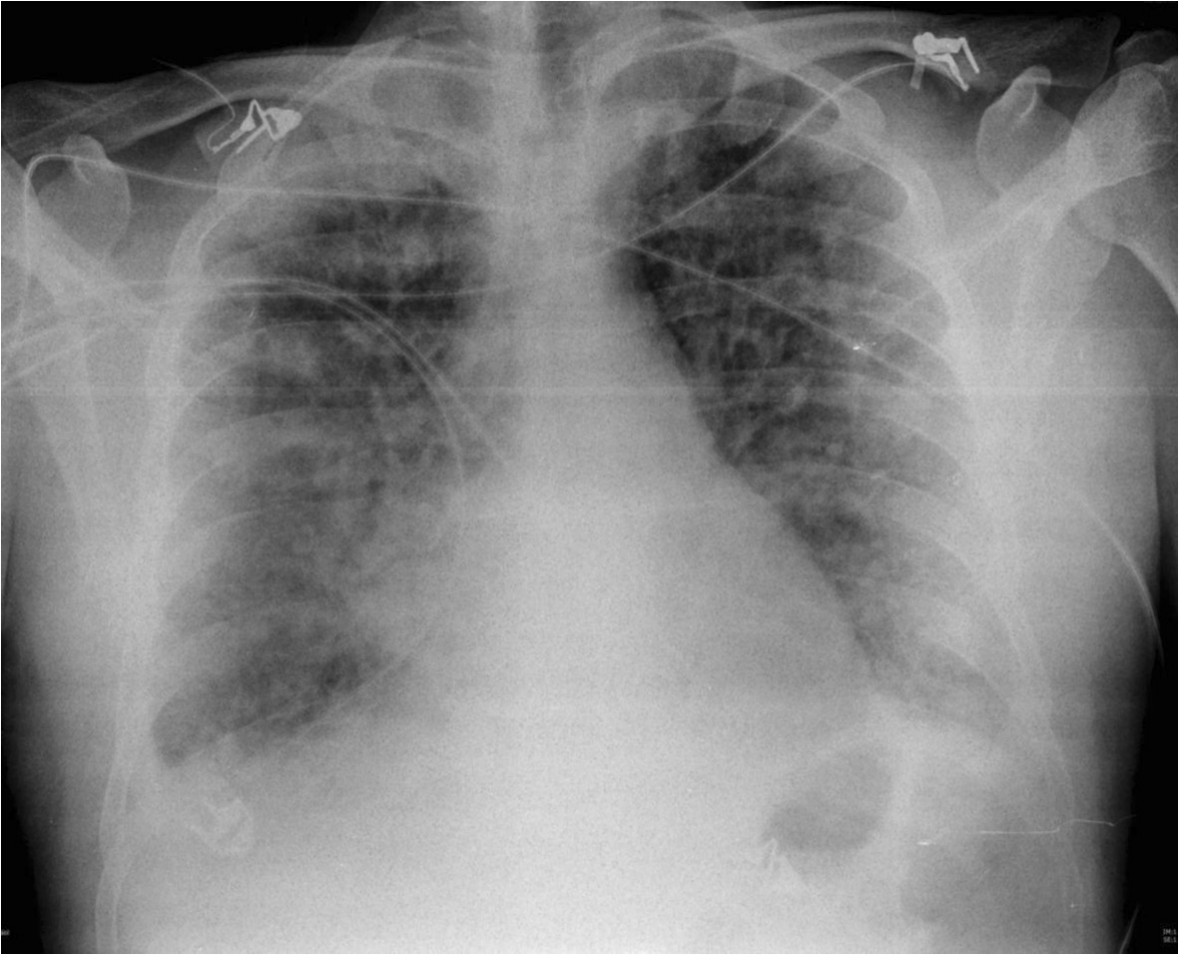
**Anteroposterior chest radiograph after nailing.** Note the diffuse bilateral infiltrates.

**Figure 6:**
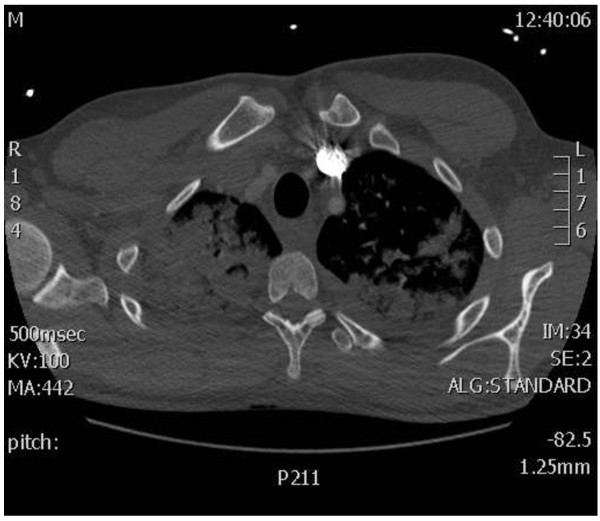
**Contrast-enhanced computed tomography scan of the chest.** Note the diffuse bilateral infiltrates in the lungs.

Standard reamed antegrade tibial nailing was performed 6 hours later (Figure 3) with fluted, flexible, intramedullary reamers to insert a 12-mm T2 nail (Stryker/Howmedica, Rutherford, NJ, USA) with proximal and distal locking screws. Just after introduction of the nail, the overall state of the patient started to deteriorate, with decreased oxygen saturation and a confusional state. When the surgical procedure had been completed, the patient was transferred to the intensive care unit (ICU), where he presented with dyspnea, hypoxemia (blood gas analysis revealed the PO_2_ to be < 60 mmHg), and fever (38.5°C). Twenty-four hours later, petechiae appeared on the lateral chest and abdomen and on the right axilla (Figure 4). Chest radiography and contrast-enhanced computed tomography (CT) showed diffuse bilateral pulmonary infiltrates (snow-storm appearance) (Figures 5 and 6).

During his 5-day stay in the ICU, the patient did not require intubation, although supplemental oxygen was provided (initially, 4 liters/min via nasal cannulae) under continuous pulse oximetry monitoring and repeated blood gas analyses until complete resolution of his symptoms. After 1 week the patient was asymptomatic, and his chest radiograph was normal. Echocardiography performed before hospital discharge showed no evidence of cardiac involvement that could explain a paradoxical fat embolism [4]. Outpatient follow-up was performed until the fracture healed completely and uneventfully.

## Discussion

Fat embolism is usually diagnosed on the basis of clinical findings [1]– [3]. According to the literature, Gurd’s criteria [1], consisting of major and minor clinical features, is the most commonly used diagnostic tool. Up to now, the diagnosis of FES has remained clinical [3] without a reference gold standard system. Our patient showed the pathognomonic triad of petechiae, hypoxia, and confusion. Radiological and CT findings confirmed the diagnosis.

Reamed intramedullary nailing continues to be the gold standard for stabilizing femoral and tibial shaft fractures. Reaming of the femoral or tibial canal allows insertion of a larger-diameter nail, optimizing the mechanical environment. Also, with the release of the products of reaming (endogenous growth factors and reaming debris), it provides an osteogenic stimulus. It is known, however, that reaming the medullary canal stimulates the immunoinflammatory system, leading to a “second hit” phenomenon. This is especially true in patients with a high ISS and associated chest injuries [13,14].

In an attempt to reduce the incidence of fat embolism during reaming and nailing of long bone fractures, the reamer/irrigator/aspirator (RIA) system was developed (Synthes, Paoli, PA, USA). Experimental data suggest that the RIA device prevents fat embolism, although clinical evidence is lacking [15].

From a pathological point of view, fat emboli occur in nearly all patients after long bone fractures, although in most patients they are benign and without clinical consequences [2]. The reason for the development of FES in some individuals and not others remains unclear.

Based on the ISS, Pape *et al.*[16] categorized patients with femoral fractures as being stable, borderline, unstable, or in extremis. Because our patient was clinically categorized as stable (ISS of 9, thoracic Abbreviated Injury Score of 0, NISS of 9), we performed the nailing procedure. From an immunological point of view, our patient probably was not stable. He was likely borderline stable/unstable.

## Conclusion

FES can occur during nailing even in a stable patient with an isolated open tibial fracture. This case report involved the knowledge gained from several clinical medical specialties, including traumatology, anesthesiology, neumology, and intensive care. Anyone involved in treating patients who present with a long bone fracture and sudden respiratory impairment should keep in mind the possibility of FES.

## Consent

Written informed consent was obtained from the patient for publication of this case report and accompanying images. A copy of the written consent is available for review by the Editor-in-Chief of this journal.

## Abbreviations

CT: Computed tomography; FES: Fat embolism syndrome; ICU: Intensive care unit; ISS: Injury severity score; NISS: New injury severity score.

## Competing interests

The authors declare that they have no competing interests.

## Authors’ contributions

GA made substantial contributions to the conception and design of this study, including collection of data, literature review, analysis, and drafting the manuscript. IS participated in the design of this study and helped draft the manuscript. LL-D contributed to the conception and participated sufficiently in the work to take responsibility for the content. All of the authors read and approved the final manuscript.
